# Development of dual-function ELISA for effective antigen and antibody detection against H7 avian influenza virus

**DOI:** 10.1186/1471-2180-13-219

**Published:** 2013-10-02

**Authors:** Fang He, Mookkan Prabakaran, Yunrui Tan, Kartigayen Indira, Subaschandrabose Rajesh Kumar, Jimmy Kwang

**Affiliations:** 1 Animal Health Biotechnology, Temasek Life Sciences Laboratory, 1 Research Link, National University of Singapore, Singapore 117604, Singapore; 2 Department of Microbiology Faculty of Medicine, National University of Singapore, Singapore, Singapore

**Keywords:** H7 AIV, Dual function ELISA, Surveillance in poultry

## Abstract

**Background:**

Outbreaks in poultry involving influenza virus from H7 subtype have resulted in human infections, thus causing a major concern for public health, as well as for the poultry industry. Currently, no efficient rapid test is available for large-scale detection of either antigen or antibody of H7 avian influenza viruses.

**Results:**

In the present study, a dual function ELISA was developed for the effective detection of antigen and antibody against H7 AIVs. The test was established based on antigen-capture-ELISA and epitope blocking ELISA. The two Mabs 62 and 98 which were exploited in the assay were identified to recognize two conformational neutralizing epitopes on H7 HA1. Both of the epitopes exist in all of the human H7 strains, including the recent H7N9 strain from China and > 96.6% of avian H7 strains. The dual ELISA was able to detect all of the five H7 antigens tested without any cross reaction to other influenza subtypes. The antigen detection limit was less than 1 HA unit of H7. For antibody detection, the sensitivity and specificity of the dual ELISA was evaluated and compared to HI and microneutralization using immunized animal sera to different H7 strains and different subtypes of AIVs. Results indicated that antibodies to H7 were readily detected in immunized animal sera by the dual ELISA whereas specimens with antibodies to other AIVs yielded negative results.

**Conclusions:**

This is the first dual-function ELISA reported for either antigen or antibody detection against H7 AIVs. The assay was highly sensitive and 100% specific in both functions rendering it effective for H7 diagnosis.

## Background

Recurrence of highly pathogenic avian influenza (HPAI) virus subtype H7 in humans and poultry continues to be a serious concern to public health. Before 2002, only occasional case reports of human H7 influenza virus infections occurred as a result of direct animal-to-human transmission or laboratory accidents and most of these infections resulted in conjunctivitis and/or mild influenza-like illness [1]. In 2003, an HPAI H7N7 outbreak in the Netherlands infected 89 people who were in close contact with affected poultry, including one fatal case, and led to the culling of over 30 million birds [2]. The most recent outbreak of H7N9 strains in China resulted in more than 130 human cases, including 36 deaths, making H7 subtype HPAI viruses the focus of public attention [3]. WHO has listed HPAI H7N9 as one of the most lethal viral pathogens [4]. Most of the infected patients had a history of poultry contact, indicating the transmission from poultry to human. The scale of poultry outbreaks and its association with cases of human infection with H7 viruses highlights the need for efficient diagnosis and continued surveillance of this virus subtype [5].

Conventional laboratory methods for influenza virus detection include virus isolation in embryonated eggs or Madin-Darby canine kidney (MDCK) cells, followed by subsequent HA subtype identification using serological methods. Molecular detection methods such as real-time PCR assays have been widely applied for the laboratory diagnosis of influenza infections [6,7] and HA subtype identification [8]. However, both conventional and laboratory methods are technically demanding and are not suitable for on-site use in field investigations. The development of rapid H7 subtype influenza virus detection tests in dot ELISA (enzyme-linked immunosorbent assay) [9], AC-ELISA (antigen-capture ELISA), and chromatographic strip formats [10] using H7 monoclonal antibodies (MAbs) is hence preferred.

Serological investigations to detect specific antibodies from H7 infection in poultry and humans are critical to the success of disease prevention and control programs. However, due to the lack of a specific and sensitive monoclonal antibody, there are no serologic tests available against H7 AIV. Microneutralization is currently used as the “gold standard” for subtyping. However, the test is labor-intensive and its sensitivity is limited, rendering it impractical for rapid and high-throughput diagnostics. The HI test and indirect ELISA are considered to be simple serology tests. However, low sensitivity and subtype cross-reactivity significantly limit the value of these assays [11]. Competitive ELISAs (cELISA), also called epitope blocking ELISAs, are widely used for serological detection of antibodies to influenza viruses [12], mainly due to their sensitivity and simplicity. The cELISA makes it possible to provide general assays for testing sera from different avian species, humans, and other species without changing any of the test reagents [13].

It is a challenge to combine AC-ELISA and cELISA on the same plate with the same amount of antibodies. The selected Mabs are required to target conserved antigenic epitopes and compete to host antibodies in infected sera for the epitope binding. In this study, two H7 Mabs were identified to meet these requirements and assembled in a dual-function-ELISA for universal H7 diagnosis via either antigen or antibody detection. The sensitivity and specificity for both functions were evaluated. The results indicated that for the first time, antigen and antibody detection could be performed with the same device and Mabs for specific and sensitive H7 AIV detection.

## Methods

### Ethics statement

All animal experiments were carried out in accordance with the Guidelines for Animal Experiments of the National Institute of Infectious Diseases (NIID). Experimental protocols were reviewed and approved by Institutional Animal Care and Use Committee of the Temasek Life Sciences Laboratory, National University of Singapore, Singapore. (IACUC approval number TLL-10-012).

All experiments involving human H7 strains were performed in a biosafety level 3 (BSL-3) containment laboratory in compliance with CDC/NIH and WHO recommendations and were approved by the Agri Veterinary Authority (AVA) of Singapore.

### Viruses and cell lines

The viruses used were listed in Table 1. H7N1 (A/Chicken/Malaysia/94) and part of other non-H7 AIV strains were obtained from the Agri-Food and Veterinary Authority of Singapore. Reassortant influenza virus H7N3 (A/Canada/rv504/04), H7N6 (A/quail/Aichi/3/09), H7N7 (A/duck/Hokkaido/1/10), H7N7 (A/Netherlands/219/03), H2, H6, H8, H11-H13, H5N1 (A/Vietnam/VN1203/03/) and H1N1 (A/TLL51/Singapore/09) were generated by reverse genetics as described previously [14]. Briefly, the complementary DNA of the HA and NA genes of influenza viruses were synthesized based on the sequences from the NCBI influenza database while the six cDNAs of the internal genes were synthesized based on the PR8 (A/Puerto Rico/8/1934) virus sequence (GenScript, USA). The cDNA of each of the eight influenza virus gene segments was inserted between the pol I promoter (pIh) and the pol I terminator of pClpolsaplT vector (kindly provided by Ruben Donis, CDC, USA) and cotransfected into cocultured 293 T human embryonic kidney cells and Madin–Darby canine kidney (MDCK) cells using lipofectamine 2000 (Life Technologies, USA). MDCK cells were maintained in Dulbeccos Modified Eagle Medium (DMEM; Life Technologies, USA) containing 10% Fetal Bovine Serum (FBS; Life Technologies, USA). 293 T were maintained in Opti-MEMI (Life Technologies, USA) containing 5% FBS. After 48 h the transfected supernatants were collected and virus titers were determined by standard hemagglutination assays. The sequences were confirmed using a specific set of universal primers as described previously (21). Viruses were propagated in 10 day old specific pathogen free embroyonated chicken eggs at 37°C. The tissue culture infectious dose 50 (TCID_50_) of reassortant virus was then calculated by the Muench-Reed method (1938).

**Table 1:** HI and neutralization (VN) titer of 62 and 98 (200 ug/ml) against different H7

**Virus**	**Subtype**	**HI titer**	**VN titer**
		**(Mab 62, 98)**	**(Mab 62, 98)**
A/Chicken/Malaysia/94*	H7N1	256, 256	640, 640
A/Canada/rv504/04	H7N3	128,256	320, 640
A/quail/Aichi/4/09	H7N6	64, 64	80, 80
A/duck/Hokkaido/1/10	H7N7	128, 256	320, 640
A/Netherlands/219/03	H7N7	256, 256	640, 1280
A/Shanghai/1/13*	H7N9	64, 128	160, 320
A/Puerto Rico/8/34	H1N1	<8, <8	<20, <20
A/TLL51/Singapore/09	H1N1	<8, <8	<20, <20
A/duck/Nanchang/4-184/2000	H2N9	<8, <8	<20, <20
A/Chicken/Malaysia/02*	H3N2	<8, <8	<20, <20
A/Chicken/Malaysia/92*	H4N1	<8, <8	<20, <20
A/Vietnam/VN1203/03	H5N1	<8, <8	<20, <20
A/Shorebird/DE/12/04	H6N8	<8, <8	<20, <20
A/duck/Yangzhou/02/05	H8N4	<8, <8	<20, <20
A/chicken/Malaysia/98*	H9N2	<8, <8	<20, <20
A/mandarin duck/Malaysia/98*	H10N5	<8, <8	<20, <20
A/pintail/Alberta/84/2000	H11N9	<8, <8	<20, <20
A/pintail/Alberta/49/03	H12N5	<8, <8	<20, <20
A/gull/Maryland/704/1977	H13N6	<8, <8	<20, <20

HI titer below 8 and VN titer below 20 indicated negative activity. *: wild type virus.

### Production and characterization of Mab

BALB/c mice were immunized twice subcutaneously at intervals of 2 weeks with BEI (binary ethylenimine) inactivated H7N1 (A/Chicken/Malaysia/94) and adjuvant (SEPPIC, France). Mice were boosted with the same viral antigen, 3 days before the fusion of splenocytes with SP2/0 cells [15]. The fused cells were seeded in 96-well plates, and their supernatants were screened by immunofluorescence assays as described below. The hybridomas that produced the Mabs were cloned by limiting dilution at least three times. The positive Mabs were tested for their hemagglutination inhibition activity as described below. Immunoglobulins from selected positive Mabs were isotyped using a commercial isotyping kit (Amersham Bioscience, England) as described in the manufacturer’s protocol. The hybridoma suspension was harvested 3 days postseeding and cell debris pelleted by centrifugation at 400 g for 10 min, followed by collection of the supernatant and storage at -20°C. Mabs were purified with Montage kit Prosep-G (Millipore) for IgG.

### Experimental serum samples

Inactivated AI viruses (Table 1) were emulsified in ISA-70 (SEPPIC, France) adjuvant and injected intramuscularly to the groups of three weeks old white leghorn chickens (n = 4). The booster was given twice at two-week intervals. Sera were prepared from the blood collected 10 days after 1st injection and 2nd injection. Antibody responses to the homologous strains were evaluated by HI as described below. Groups of mice (n = 4) were injected intramuscularly with different inactivated H7 AIVs (Table 1) individually emulsified in adjuvant (SEPPIC, France). The injections were repeated twice at two-week intervals. In addition, guinea pigs were immunized with inactivated H7N1 (A/Chicken/Malaysia/94). Blood was collected 14 days after the 2nd immunization.

### Hemagglutination inhibition assay

Hemagglutination inhibition (HI) assays were performed as described previously [16]. Briefly, Mabs were serially diluted (2 fold) in V-bottom 96-well plates and mixed with 4 HA units of H7 virus. Plates were incubated for 30 min at room temperature, and 1% chicken RBCs were added to each well. The hemagglutination inhibition endpoint was the highest Mab dilution in which agglutination was not observed.

### Isolation and analysis of escape mutants

The epitope recognized by Mab 62 was mapped by characterization of escape mutants as described previously [9]. Briefly, H7N1 parental viruses were incubated with an excess of Mab for 1 h and then inoculated into 11 day old embryonated chicken eggs. The eggs were incubated at 37°C for 48 h. Virus was harvested and used for cloning in limiting dilution in embryonated chicken eggs and the escape mutants were plaque purified. The HA gene mutations were then identified by sequencing and comparison with the sequence of the parental virus.

### Microneutralization assay

Neutralization activity of Mab against H7 strains was analyzed by microneutralization assay as previously described [17]. Briefly, Mab was serially two-fold diluted and incubated with 100 TCID50 of different clades of H7 strains for 1 h at room temperature and plated in duplicate onto MDCK cells grown in a 96-well plate. The neutralizing titer was assessed as the highest Mab dilution in which no cytopathic effect was observed by light microscopy.

### H7 baculovirus production

The recombinant baculovirus vector was generated as described previously [18]. The full length HA gene was amplified from H7N7 (A/NL/219/03) reassortant virus in a standard PCR reaction. The amplified HA gene was inserted into the shuttle vector pFASTBacHT A (Invitrogen, San Diego, CA, USA) for expression under the white spot syndrome virus (WSSV) immediate early (ie1) promotor. This expression cassette was integrated into the baculovirus genome within DH10BacTM (Invitrogen, USA) through site specific transposition according to the protocol of the Bac-to-Bac system (Invitrogen). SF9II cells were maintained in SF900II serum free medium (Gibco BRL, USA) at 28°C for recombinant baculovirus synthesis. The recombinant bacmid was then transfected into SF9II cells and the supernatant containing recombinant baculovirus displayed H7-HA (Bac-H7) was harvested at 96 h post-infection.

### Dual-function-ELISA

96-well, round-bottom microtiter plates (Nunc, Roskilde, Demark) were coated with 0.5 ug/well of capture MAb 98 in 100 ul of carbonate buffer (73 mM sodium bicarbonate and 30 mM sodium carbonate, pH 9.7) overnight at 4°C or 37°C for 2 h. The plates were washed twice with PBST, followed by two washes with PBS after each incubation with antibody or antigen. The antibody-coated plates were blocked by incubation with 100 ul of blocking buffer (PBS containing 5% milk) for 1 h at room temperature. For antigen detection, the blocked plates were then incubated at 37°C for 1 h with 100 ul of virus-containing samples diluted in PBST. For antibody detection, 50 ul of serum samples mixed with 50 ul of H7 surface expressing baculovirus of 8 HAU were added to the blocked plates for 1-hour-incubation at 37°C. Virus binding or antibody blocking was detected by incubation for 1 h at 37°C with 100 ul of horseradish peroxidase-conjugated detection MAb 62 (800 ng) (in-house labeling; Roche). Chromogen development was mediated by the addition of 100 ul of freshly prepared substrate solution (o-phenylenediamine-dihydrochloride; Sigma). The reaction was stopped with sulfuric acid of 0.1 N, and the optical density at 490 nm was recorded. The antigen detection limit was determined by the optical density value that gave a signal-to-noise ratio of 3. For antibody detection, the OD intensity reduction caused by serum antibodies blocking Mab binding was calculated for each sample dilution by using the formula: % inhibition = [(negative reference serum OD-test serum OD)/(negative reference serum OD-positive reference serum OD)]×100%. To determine the cut-off value of antibody detection, specific pathogen-free chicken sera, mice and guinea pigs were obtained from the Animal Health Biotechnology Serum Bank, Temasek Life Sciences Laboratory, Singapore.

## Results

### Mab 62 and 98 recognize conserved neutralizing epitopes on H7 AIVs

A panel of Mabs against influenza hemagglutinin was screened for efficient recognition of different strains of H7 viruses. Based on the results of the HI assay and virus neutralization (Table 1), Mab 62 and 98 were selected for further studies due to their high HI activity against a wide range of H7 viruses from birds and humans, including strains from the recent H7N9 outbreak in eastern China. Both the Mabs belong to the IgG1 isotype. The virus neutralizing activity of Mab 62 and 98 was further confirmed to be positive against H7 AIVs. Based on this, the amino acids involved in forming the epitope of Mab 62 and 98 were analyzed using selection of neutralization escape mutants. A/chicken/Malaysia/94 H7N1 virus was used as parental virus for the selection. Sequences of the complete HA genes isolated from multiple escape variants were compared with the parental virus. It was found that mutants generated with Mab 62 have a single mutation on amino acid 175 from Lysine to Glutamate. Mab 98 carried mutations either at amino acid 136 (Ser to Gly), or 137 (Gly to Arg). The numbering of amino acid on HA starts from “ATG” and includes the signal peptide.

In order to determine the significance of the neutralization epitopes of Mab 62 and 98, the protein polymorphism of H7 was studied (Table 2), taking into account all H7 sequences in the NCBI database. On the 175th amino acid, Lysine and Asparagine appear in more than 99.9% of H7 AIV strains listed. Lysine is the most dominant amino acid with the frequency of 97.9% among avian H7 strains and 100% among human H7s. On the 136th amino acid, Serine exists in 96.6% of avian strains and 100% of human H7 strains, while Glycine on the 137th amino acid exists in 99.9% of avian H7 and 100% of human strains. This finding indicates that the two Mabs are able to recognize or neutralize all the H7 human strain identified so far, suggesting their potential for universal H7 AIV detection.

**Table 2:** Epitope frequency in both human and avian H7 strains

**Mab**	**Amino acid**	**Human frequency**	**Avian frequency**
98	136 Ser	100%	96.6%
	137 Gly	100%	99.9%
62	175 Lys	100%	97.9%

### Development of the dual-function-ELISA

**Figure 1:**
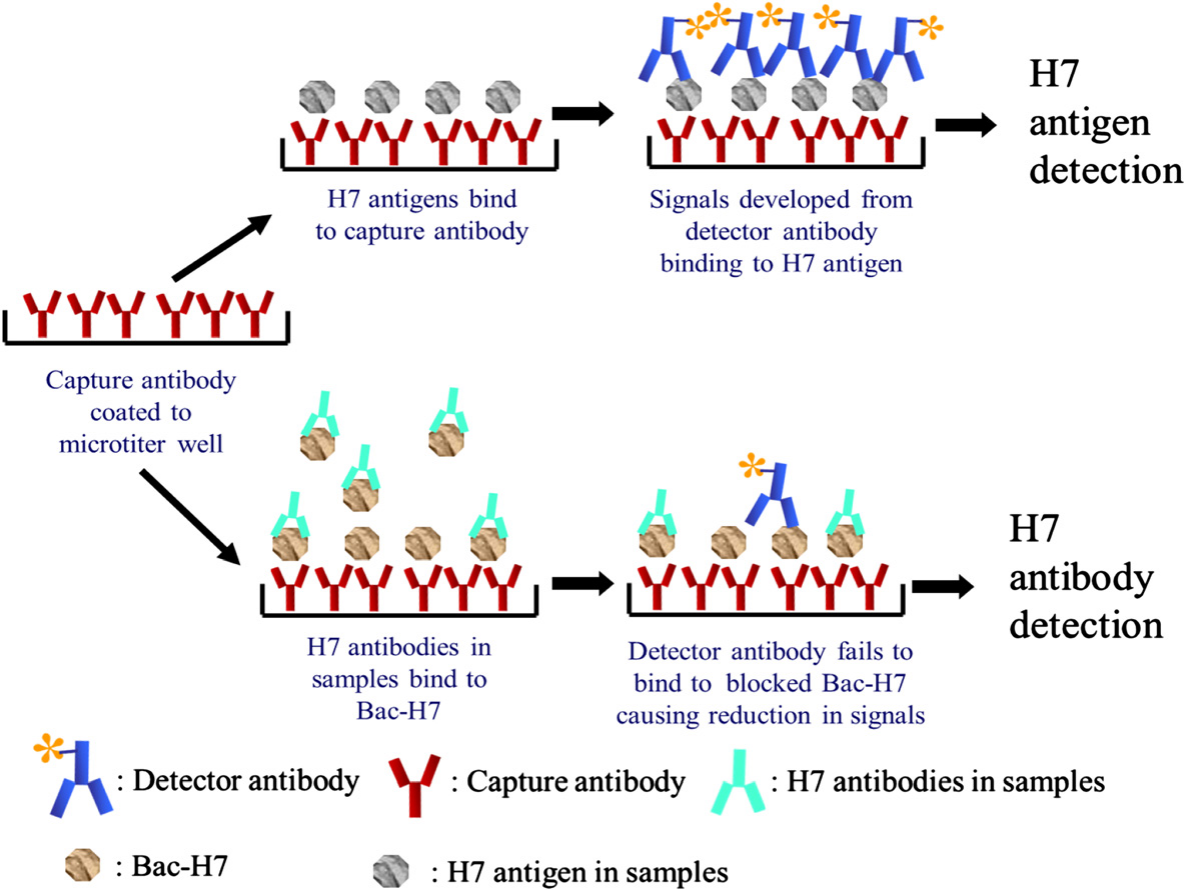
Procedures of both antigen and antibody detection in the dual-function-ELISA.

The dual-function-ELISA was operated as shown in Figure 1. H7 antigen can be detected in an AC-ELISA based on H7 specific Mabs. Mab 62 was randomly selected as the detector antibody and Mab 98 was used as the capture antibody due to their equivalent performance in the reversible use in H7 AC-ELISA. Optimal concentrations of MAbs 62 and 98 for detection and capture were determined by two-way titration of MAb concentrations. The combination that gave the highest signal-to-noise ratio was determined to be 0.5 ug/well of capture MAb 98 and 0.9 ug/well of MAb 62 for detection. The tested virus was considered to be positive with H7 antigen in the dual ELISA when the absorbance was three times higher than that of the non-H7 viruses.

Serum antibodies to H7 can be detected by virtue of their ability to block the recognition of the target epitope by a H7 specific Mab in an ELISA assay. To combine this assay to the AC-ELISA, serum samples were incubated with the fixed amount of recombinant baculovirus, which displays H7 on the virus surface, before being loaded to the plate coated with the capture Mab. H7 antibody titers in samples were determined based on the reduction of the detected H7 baculovirus. Different concentrations of H7 baculovirus were tested before confirming the optimal concentration at 8 HAU. Serum panels from normal or H7 immunized chicken and mice were used to determine the cut-off value. First, a panel of normal serum samples from 16 chicken and 20 mice lacking antibodies to H7 was used to determine the baseline of non-specific reduction in Mab 62 binding to H7 antigen in the dual ELISA. Mean reduction of dual ELISA readings was 6.5% for this serum panel, with a standard deviation (SD) of 7.1. Specific blocking activities can be determined with 95% confidence if a “cut-off value” of ≥30% is set for serum samples. The latter was obtained by adding 3 SD to the mean 6.5% blocking (6.5 + 21.3 = 27.8%). In the test, the dilution factor of each serum sample at was recorded when it presented ≥30% signal blocking rate. Additionally, the blocking rate of each sample diluted at 20 times was recorded for comparison.

### Specificity and sensitivity of H7 antigen detection by the dual-function-ELISA

**Figure 2:**
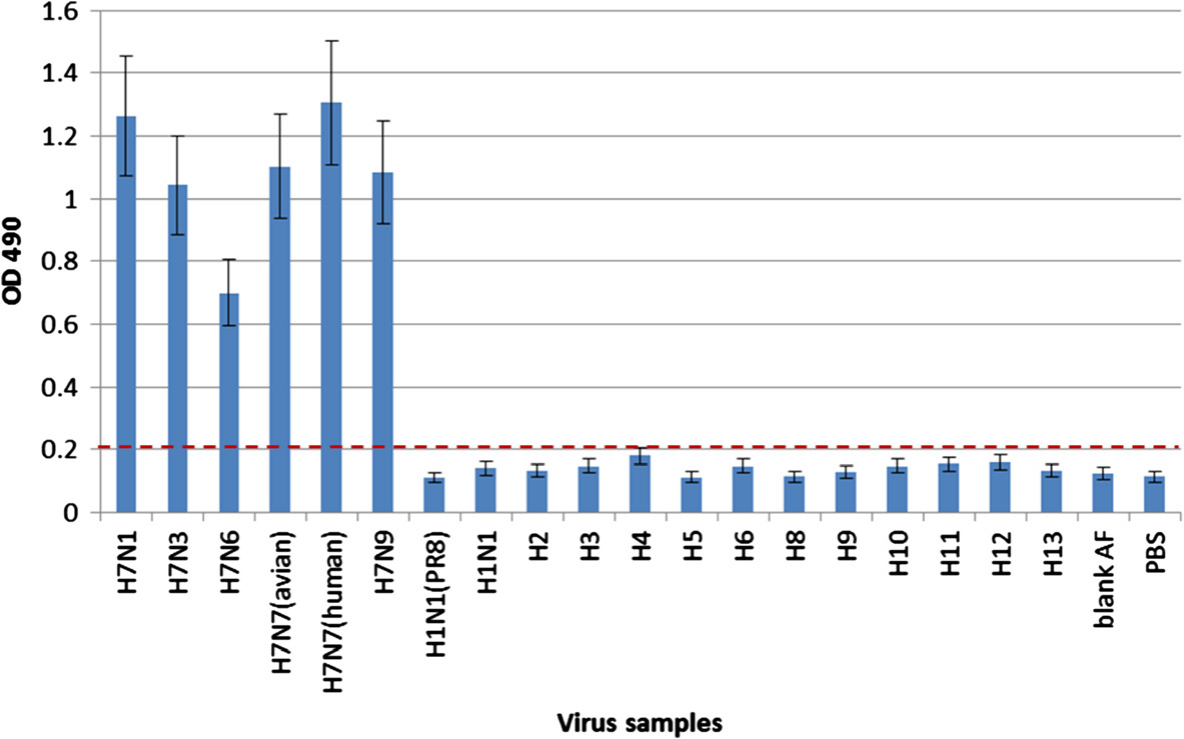
**Specificity of H7 antigen detection in the dual ELISA.** The specificity of H7 antigen detection in the dual-ELISA was examined with 100 ul of PBS containing the H7 strains adjusted to an HA titer of 8 or non-H7 viruses with HA titers of ≥16. Values represent the means of absorbances of duplicate wells from two independent tests. OD 490: optical density at 490 nm; dotted line: cut-off values; Blank AF: allantoic fluid without virus.

The specificity of H7 antigen detection by the dual ELISA was tested with 6 H7 strains from humans and avian species and 13 representative non-H7 subtype influenza virus strains from different regions and years, including pandemic influenza and avian influenza virus strains circulating in humans (Figure 2). Viruses of H7 or HA subtypes not available in our laboratory were rescued by reverse genetics with the six internal genes from A/Puerto Rico/ 8/34. The reactivity and specificity of H7 antigen detection in the dual-ELISA were examined with 100 ul of PBS containing the H7 strains adjusted to an HA titer of 8. Non-H7 viruses with HA titers of ≥16 were used in order to eliminate false-positive results. No cross-reactivity was observed for any of the non-H7 subtype viruses tested.

**Figure 3:**
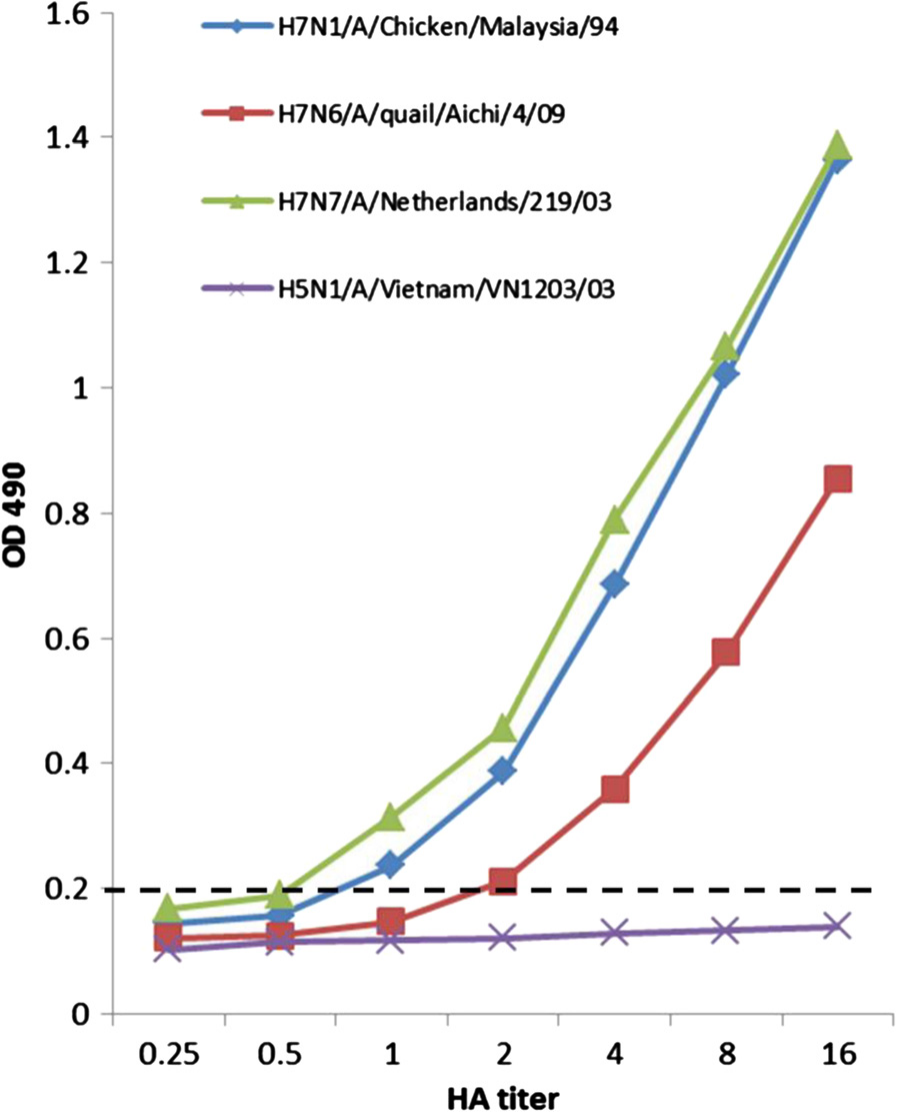
**Sensitivity of H7 antigen detection in the dual ELISA.** Tested viruses with an HA titer of 16 were twofold diluted and subjected to the dual ELISA for antigen detection. Values represent the means of absorbance of duplicate wells from two independent tests. OD 490: optical density at 490 nm; dotted line: cut-off values.

The analytical sensitivity of H7 antigen detection in the dual ELISA was determined against four different H7 strains which had absorbance readings ranging from 0.7 to 1.3 at 8 HAU (Figure 3). The three selected H7 viruses were diluted serially for the determination of the detection limit based on virus HA titer. With a cut-off value of 0.2, the detection limit was determined to be 100 ul of sample containing 1 HA titer of virus (equal to TCID_50_ 10^3.2^ of H7N7 A/Netherlands/219/03; TCID_50_ 10^2.12^ of H7N1 A/Chicken/Malaysia/94) for viruses that had average and higher-than-average absorbance, while it was 2 HA titers (equal to TCID_50_ 10^2.354^ of H7N6 A/quail/Aichi/4/09) for viruses that had lower-than-average absorbance. The detection limit of HI test for influenza virus was determined at 2 HAU (100 ul) and subtype cross-reactivity were observed.

### Specificity of H7 antibody detection by the dual-function-ELISA

**Figure 4:**
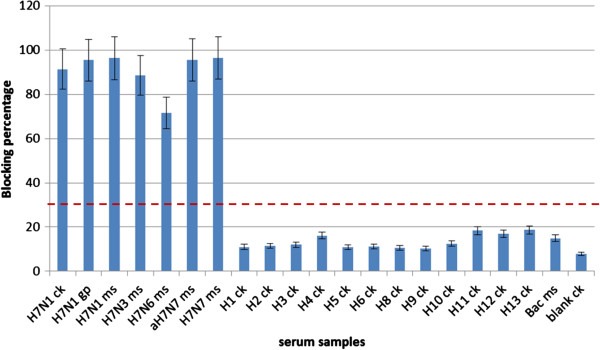
**Specificity of H7 antibody detection in the dual ELISA.** Sera from different animals immunized with different subtypes of influenza viruses were collected 10 days after the 2nd immunization and normalized to a HI titer of 16 before tested in the dual ELISA. Inhibition above the cut-off value of 30% blocking was considered as positive; i.e. antibodies to H7 were present. The results were expressed as the arithmetic mean of percent blocking values. aH7N7: A/duck/Hokkaido/1/10; Ck: chicken; Gp: guinea pig; Ms: mouse; Bac: wildtype baculovirus immunized serum; Blank: preimmune serum. Dotted line: cut-off values.

The specificity of the H7 detection by the dual ELISA was investigated using a panel of antisera from experimentally immunized chickens, mice and guinea pigs. Animal sera collected 10 days after the 2nd immunization were first diluted to obtain HI titer of 16 to the homologous virus to normalize antibody concentrations prior to use in EB-ELISA. Sera from chicken immunized with H7N1 influenza viruses (Figure 4) presented ≥85% inhibition in Mab 62 binding, while sera from chickens immunized with H1-H6 and H8-H13 showed maximum blocking of 10%, well below the 30% threshold established for samples containing H7 specific antibodies. No inhibition was detected with sera immunized with wild type baculovirus. Positive inhibition was also observed with all mouse sera from individual immunizations with 4 different H7 strains, indicating the assay is specific to detect H7 antibodies. All animal sera from H7 immunization, including chicken, mouse and guinea pigs, showed positive blocking in the dual ELISA, indicating the assay is effective for sera from any species. These results indicate that the antibody detection in the dual ELISA could positively identify serum samples containing antibodies to H7 without any cross reaction to sera from other subtypes.

### Sensitivity of H7 antibody detection by the dual-function-ELISA

The sensitivity of H7 antibody detection in the dual ELISA was primarily determined by comparison to virus neutralization and HI using purified Mab 62. As shown in Table 3, in the dual ELISA, 40 ng of Mab 62 was sufficient to reach the endpoint corresponding to a blocking rate of more than 30%, while at least 160 ng of the same Mab 62 was needed to neutralize 100 TCID50 of H7N7 (A/Netherlands/219/03) virus or inhibit hemagglutination. Additional comparisons of the dual ELISA and virus neutralization in antibody detection were made using H7 immunized mice sera (Table 4). The neutralization titers of mice sera after only one immunization with variant H7 AIV strains individually ranged from 40 to 320 against H7N7 (A/Netherlands/219/03). The same batch of sera was tested in the dual ELISA where the endpoint titers ranged from 100 to 1000. No positive activity was detected for pre-immunization serum samples by either test. The comparison indicated that the dual ELISA was able to detect a lower concentration of H7 specific antibody and present a higher signal titer than virus neutralization.

**Table 3:** The detection limits of the dual ELISA in antibody detection

**EB-ELISA**		**Microneutralization**^**a**^		**HI**^**a**^	
**Mab amount**	**Inhibition rate**	**Mab amount**	**Titer**	**Mab amount**	**Titer**
5 ug	92.6%	5 ug	640	5 ug	256
1 ug	64.87%	1.25 ug	160	1.25 ug	64
0.2 ug	48.99%	0.313 ug	40	0.313 ug	16
***0.04 ug***^***b***^	***31.05%***	***0.16 ug***^***b***^	***20***	***0.16 ug***^***b***^	***8***
0.008 ug	12.84%	0.08 ug	<20	0.08 ug	<8

^a^HI and microneutralization assay based on a neutralizing Mab.

^**b**^The detection limit of each test is indicated in bold and italics format.

**Table 4:** Comparison between the dual-function-ELISA and virus neutralization in antibody detection with pooled mice sera after a single H7 immunization

**Virus immunized**	**Inhibition in dual ELISA at 1:20 dilution**	**Dual ELISA titer at 30% cut-off**	**Virus neutralization titer**
H7N3/A/Canada/rv504/04	91.47%	500	160
H7N6/A/quail/Aichi/4/09	61.64%	100	40
H7N7/A/duck/Hokkaido/1/10	92.84%	500	160
H7N7/A/Netherlands/219/03	94.68%	1000	320
Pre-immunization sera	4.14%	<20	<20

## Discussion

Increasing numbers of human infection and deaths caused by H7N9 HPAI virus are currently reported in China, making H7 subtype influenza virus one of the most threatening flu pathogens. Successful control of H7 HPAI viruses requires early virus detection and active serological surveillance of animals and humans. Despite the advantages of conventional methods such as real time PCR with high sensitivity and virus neutralization with high specificity in influenza diagnosis, the main drawback of these methods is their impracticality for field investigation. In this study, a dual-function-ELISA was developed to detect H7 AIVs by the combination of AC-ELISA and blocking ELISA. The method allows the specific and sensitive detection of both antigen and antibody of H7 AIVs with the same type and amount of monoclonal antibodies. The dual-function-assay for H7 antigen and antibody detection provides a promising prototype for a rapid test in an ever simplified format.

A specific and sensitive immunological assay relies on good monoclonal antibodies. Both Mab 62 and 98 are ofthe IgG1 isotype, which is optimal for large-scale production and purification. The relevant amino acids in the epitopes of Mab 62 and 98 were identified by the sequencing of escape mutants. The identified amino acids exist in all of the human H7 strains, including the one from the recent outbreak in China, as confirmed with virus neutralization and HI. The site targeted by Mab 98 is within the 120-loop, a part of the receptor binding site (RBS) [19] of H7, while Mab 62 recognizes an epitope located between the 180-helix and 140-loop of H7 HA1. The 180-helix is also part of the RBS and the 140-loop contributes to the recognition of RBS [20]. Targeting to the RBS makes Mab 98 a neutralizing antibody with strong affinity to H7. The amino acid position 175 for Mab 62 is close to the H7 RBS but not within it. This allows the amino acid to be conserved in neutralizing epitopes.

Most patients infected with H7N9 HPAI viruses had a history of poultry contact [21]. However, most avian species carrying infectious H7N9 viruses are asymptomatic [22]. Symptoms from H7N9 infection developed rapidly and treatments are effective only when administrated within 5 days after the onset of the symptoms [23,24]. Therefore, detection of H7 antigens at the earliest stage of infection is of crucial significance to identify infections and reduce mortality in patients. This poses a serious threat to public health and highlights the need for H7 diagnosis. Further, less cases of avian infection with H7N9 were reported than human cases, suggesting there may be other reasons for human infection besides poultry contacts. Serological assays are able to identify the history of mild or asymptomatic infection in avian species or humans, providing critical information for surveillance studies [25]. Hence, efficient serological detection in birds and humans is also important to control and study H7 HPAI viruses. It was found previously that poultry species carrying AIV antibodies are shedding less virus than SPF poultry upon asymptomatic AIV infection or infection with mild microscopic lesions [26]. Therefore, ideally, diagnostic results of both antigen and antibody detection should be consulted together to create a better understanding of H7 infection among populations. However, applying those high-tech diagnosis tests, such as Real time PCR and virus neutralization, to routine screening in public populations and birds is neither practical nor cost effective due to the limited availability of equipment and trained manpower. User-friendly rapid tests, such as dot ELISA and lateral flow, are preferred in field investigation and clinical diagnosis in the neighborhood [10,27]. All these immuno-tests are initially developed from an ELISA assay based on monoclonal antibodies [9]. In the current study, AC-ELISA and competitive ELISA were combined to a dual ELISA with standardized Mabs for both H7 antigen and antibody detection. High specificity and sensitivity were confirmed for either function in the dual ELISA against H7 AIVs. Sensitivity of antigen detection is higher than HA tests and antibody ELISA detects less H7 antibodies than conventional virus neutralization. The combination of two functions in one plate paves the way for an ever simplified rapid test. For antibody detection, the dual ELISA is even easier to prepare than conventional competitive ELISAs. A small amount of baculovirus expressed H7 antigen is sufficient for antibody blocking in the dual ELISA while highly purified and concentrated H7 antigen is required for coating in other cELISAs to minimize unspecific blocking effects [12].

## Conclusions

For the first time, the dual-function ELISA presented in this study was proven to provide a fast, simple and cost-effective platform for both antigen and antibody detection. The assay was highly sensitive and 100% specific in both functions rendering it effective for H7 diagnosis. Exploiting H7 specific neutralizing Mab 62 and 98 makes the H7 dual ELISA a more specific and sensitive assay as compared to conventional immunological tests. A rapid test based on this H7 dual ELISA will serve as effective tools for both H7 diagnosis and surveillance investigation, meeting the needs to counter the ongoing outbreak of H7N9 in China.

In conclusion, the dual-function ELISA presented in this study was proven to provide a fast, simple and cost-effective platform for both antigen and antibody detection. Exploiting H7 specific neutralizing Mab 62 and 98 makes the H7 dual ELISA a more specific and sensitive assay as compared to conventional immunological tests. A rapid test based on this H7 dual ELISA will serve as effective tools for both H7 diagnosis and surveillance investigation, meeting the needs to counter the ongoing outbreak of H7N9 in China.

## Competing interests

The authors declare that they have no competing interests.

## Authors’ contributions

Conceived and designed the experiments: FH, JK. Performed the experiments: FH, YT, KI. Analyzed the data: FH JK. Contributed reagents/materials/analysis tools: MP, SRK. Wrote the paper: FH. All authors read and approved the final manuscript.

## References

[B1] Belser JA Bridges CB Katz JM Tumpey TM Past, present, and possible future human infection with influenza virus A subtype H7 Emerg Infect Dis 2009 13 6 859 865 10.3201/eid1506.090072 19523282 PMC2727350

[B2] Koopmans M Wilbrink B Conyn M Natrop G van der Nat H Vennema H Meijer A van Steenbergen J Fouchier R Osterhaus A Transmission of H7N7 avian influenza A virus to human beings during a large outbreak in commercial poultry farms in the Netherlands Lancet 2004 13 9409 587 593 10.1016/S0140-6736(04)15589-X 14987882

[B3] Wu S Wu F He J Emerging risk of H7N9 influenza in China Lancet 2013 13 9877 1539 1540 23602315 10.1016/S0140-6736(13)60767-9 PMC7137082

[B4] Horby P H7N9 is a virus worth worrying about Nature 2013 13 7446 399 10.1038/496399a 23619655

[B5] Malik Peiris JS Avian influenza viruses in humans Rev Sci Tech 2009 13 1 161 173 19618624 10.20506/rst.28.1.1871

[B6] Imai M Ninomiya A Minekawa H Notomi T Ishizaki T Van Tu P Tien NT Tashiro M Odagiri T Rapid diagnosis of H5N1 avian influenza virus infection by newly developed influenza H5 hemagglutinin gene-specific loop-mediated isothermal amplification method J Virol Methods 2007 13 2 173 180 10.1016/j.jviromet.2006.12.004 17218021

[B7] Corman V Eickmann M Landt O Bleicker T Brunink S Eschbach-Bludau M Matrosovich M Becker S Drosten C Specific detection by real-time reverse-transcription PCR assays of a novel avian influenza A(H7N9) strain associated with human spillover infections in China Euro Surveill 2013 13 16 23611031

[B8] Thontiravong A Payungporn S Keawcharoen J Chutinimitkul S Wattanodorn S Damrongwatanapokin S Chaisingh A Theamboonlers A Poovorawan Y Oraveerakul K The single-step multiplex reverse transcription- polymerase chain reaction assay for detecting H5 and H7 avian influenza A viruses Tohoku J Exp Med 2007 13 1 75 79 10.1620/tjem.211.75 17202774

[B9] He F Soejoedono RD Murtini S Goutama M Kwang J Complementary monoclonal antibody-based dot ELISA for universal detection of H5 avian influenza virus BMC Microbiol 2010 13 330 10.1186/1471-2180-10-330 21192824 PMC3023680

[B10] Cui S Tong G A chromatographic strip test for rapid detection of one lineage of the H5 subtype of highly pathogenic avian influenza J Vet Diagn Invest 2008 13 5 567 571 10.1177/104063870802000505 18776087

[B11] Julkunen I Pyhala R Hovi T Enzyme immunoassay, complement fixation and hemagglutination inhibition tests in the diagnosis of influenza A and B virus infections. Purified hemagglutinin in subtype-specific diagnosis J Virol Methods 1985 13 1 75 84 10.1016/0166-0934(85)90091-6 3882733

[B12] Prabakaran M Ho HT Prabhu N Velumani S Szyporta M He F Chan KP Chen LM Matsuoka Y Donis RO Development of epitope-blocking ELISA for universal detection of antibodies to human H5N1 influenza viruses PLoS One 2009 13 2 e4566 10.1371/journal.pone.0004566 19238211 PMC2642733

[B13] He F Kiener TK Lim XF Tan Y Raj KV Tang M Chow VT Chen Q Kwang J Development of a sensitive and specific epitope-blocking ELISA for universal detection of antibodies to human enterovirus 71 strains PLoS One 2013 13 1 e55517 10.1371/journal.pone.0055517 23383215 PMC3561296

[B14] Ho HT Qian HL He F Meng T Szyporta M Prabhu N Prabakaran M Chan KP Kwang J Rapid detection of H5N1 subtype influenza viruses by antigen capture enzyme-linked immunosorbent assay using H5- and N1-specific monoclonal antibodies Clin Vaccine Immunol 2009 13 5 726 732 10.1128/CVI.00465-08 19321691 PMC2681585

[B15] He F Du Q Ho Y Kwang J Immunohistochemical detection of Influenza virus infection in formalin-fixed tissues with anti-H5 monoclonal antibody recognizing FFWTILKP J Virol Methods 2009 13 1 25 33 10.1016/j.jviromet.2008.09.016 18930768

[B16] Prabhu N Prabakaran M Hongliang Q He F Ho HT Qiang J Goutama M Lim AP Hanson BJ Kwang J Prophylactic and therapeutic efficacy of a chimeric monoclonal antibody specific for H5 haemagglutinin against lethal H5N1 influenza Antivir Ther 2009 13 7 911 921 10.3851/IMP1413 19918095

[B17] He F Kwang J Monoclonal antibody targeting neutralizing epitope on h5n1 influenza virus of clade 1 and 0 for specific h5 quantification Influenza Res Treat 2013 13 360675 23533740 10.1155/2013/360675 PMC3603295

[B18] Prabakaran M He F Meng T Madhan S Yunrui T Jia Q Kwang J Neutralizing epitopes of influenza virus hemagglutinin: target for the development of a universal vaccine against H5N1 lineages J Virol 2010 13 22 11822 11830 10.1128/JVI.00891-10 20844051 PMC2977865

[B19] Nobusawa E Aoyama T Kato H Suzuki Y Tateno Y Nakajima K Comparison of complete amino acid sequences and receptor-binding properties among 13 serotypes of hemagglutinins of influenza A viruses Virol 1991 13 2 475 485 10.1016/0042-6822(91)90588-3 2024485

[B20] Yang H Carney PJ Donis RO Stevens J Structure and receptor complexes of the hemagglutinin from a highly pathogenic H7N7 influenza virus J Virol 2012 13 16 8645 8652 10.1128/JVI.00281-12 22674977 PMC3421765

[B21] Zhang W Wang L Hu W Ding F Sun H Li S Huang L Li C Epidemiological characteristics of cases for influenza A (H7N9) virus infections in China Clin Infect Dis 2013 13 4 619 620 23633108 10.1093/cid/cit277 PMC7108025

[B22] Chen Y Liang W Yang S Wu N Gao H Sheng J Yao H Wo J Fang Q Cui D Human infections with the emerging avian influenza A H7N9 virus from wet market poultry: clinical analysis and characterisation of viral genome Lancet 2013 13 9881 1916 1925 10.1016/S0140-6736(13)60903-4 23623390 PMC7134567

[B23] Koopmans M de Jong MD Avian influenza A H7N9 in Zhejiang, China Lancet 2013 13 9881 1882 1883 10.1016/S0140-6736(13)60936-8 23628442

[B24] Belser JA Zeng H Katz JM Tumpey TM Infection with highly pathogenic H7 influenza viruses results in an attenuated proinflammatory cytokine and chemokine response early after infection J Infect Dis 2011 13 1 40 48 10.1093/infdis/jiq018 21148495 PMC3086437

[B25] Velumani S Ho HT He F Musthaq S Prabakaran M Kwang J A novel peptide ELISA for universal detection of antibodies to human H5N1 influenza viruses PLoS One 2011 13 6 e20737 10.1371/journal.pone.0020737 21695200 PMC3112154

[B26] Morales AC Jr Hilt DA Williams SM Pantin-Jackwood MJ Suarez DL Spackman E Stallknecht DE Jackwood MW Biologic characterization of H4, H6, and H9 type low pathogenicity avian influenza viruses from wild birds in chickens and turkeys Avian Dis 2009 13 4 552 562 10.1637/8877-041509-Reg.1 20095156

[B27] Chen Y Xu F Fan X Luo H Ge S Zheng Q Xia N Chen H Guan Y Zhang J Evaluation of a rapid test for detection of H5N1 avian influenza virus J Virol Methods 2008 13 1–2 213 215 18793671 10.1016/j.jviromet.2008.08.013

